# Epitaxial Antiferroelectric Bi_2_O_2_S Films with Superior Photoresponse

**DOI:** 10.1021/acsami.4c22419

**Published:** 2025-03-27

**Authors:** Yong-Jyun Wang, Chuan Chuang, Chia-Chen Chung, Po-Chih Chu, Wei-Chun Lin, Jian-Wei Zhang, Yu-Lun Chueh, Zhenzhong Yang, Rong Huang, Keng-Hung Chang, Heng-Jui Liu, Hsiang-Lin Liu, Jia-Yuan Sun, Xin-Yun Chang, Hao-Che Chan, Chih-Wei Luo, Yu-Miin Sheu, Jyh-Ming Wu, Yi-Cheng Chen, Ying-Hao Chu

**Affiliations:** 1 Department of Materials Science and Engineering, National Tsing Hua University, Hsinchu 300044, Taiwan; 2 Department of Photonics, National Sun Yat-sen University, Kaohsiung 804201, Taiwan; 3 Key Laboratory of Polar Materials and Devices (MOE) and Department of Electronics, East China Normal University, Shanghai 201203, China; 4 Department of Materials Science and Engineering, National Chung Hsing University, Taichung 402202, Taiwan; 5 Department of Physics, National Taiwan Normal University, Taipei 111396, Taiwan; 6 Department of Electrophysics, National Yang Ming Chiao Tung University, Hsinchu 300093, Taiwan; 7 Institute of Physics, National Yang Ming Chiao Tung University, Hsinchu 300093, Taiwan; 8 National Synchrotron Radiation Research Center, Hsinchu 300092, Taiwan

**Keywords:** Bi_2_O_2_S, epitaxy, optoelectronics, photoresponse, 2D/Si integration

## Abstract

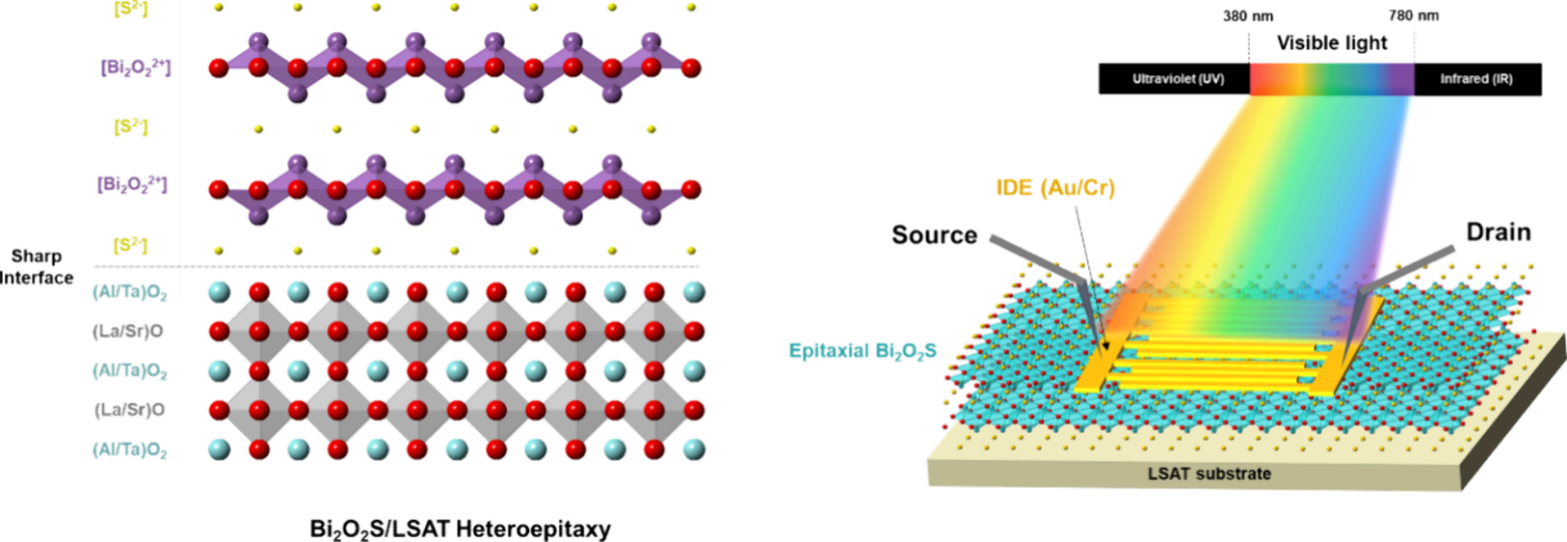

Two-dimensional bismuth oxychalcogenide is a rising material system with superior electronic properties. However, a lack of high-quality synthesis impedes the exploration of fundamental understanding and practical applications. This work presents high-quality epitaxial Bi_2_O_2_S films on (LaAlO_3_)_0.3_(Sr_2_TaAlO_6_)_0.7_ with diverse properties by taking advantage of lattice compatibility. The atomically resolved sharp interface of Bi_2_O_2_S/(LaAlO_3_)_0.3_(Sr_2_TaAlO_6_)_0.7_ heteroepitaxy is observed with the verification of centrosymmetric breaking through microscopic evidence and macroscopic characterizations. Such an epitaxial feature of the Bi_2_O_2_S film provides an essential step for applications compared to those of chemically synthesized nanomaterials. The interior polarization and piezoelectricity can be investigated through atomic-scale observation and RhB degradation of BOS. Meanwhile, this synthesized system can achieve a strong photoresponse with an on/off ratio of ∼10^4^ and a responsivity of ∼60 mA/W in the range of red light (620–750 nm). With these advantages, the demonstrated epitaxial Bi_2_O_2_S shows a huge potential for applications in high-performance optoelectronic devices.

## Introduction

Following Moore’s law, the trend of scaling down in the semiconductor industry has triggered the advancement of device performance in modern technology. Several challenges will be encountered during the minimization, such as the short-channel and tunneling effects.^1,2^ Thus, exploring new materials is crucial for improving the current situation and breaking the development bottleneck. 2D semiconductors, characterized by their atomic-scale thickness, have emerged as promising candidates for revolutionizing various electronic devices.^3−5^ Unlike conventional semiconductors, 2D materials possess unique electronic and optical properties that can be tailored to specific applications.^6−8^ Among various 2D materials, bismuth oxysulfide (Bi_2_O_2_S, BOS) is a novel 2D material with several intriguing properties. Previous studies^9−12^ have reported that BOS exhibits excellent optoelectronic properties. The suitable band gap (∼1.2 eV) enables BOS to absorb visible light effectively and transform light energy into electricity, invoking the development of a photodetector. Moreover, its high chemical stability is promising for photochemistry-related reactions,^13−16^ offering a persistent effect in those extreme conditions. On the other hand, researchers^17,18^ have discussed the existence of ferroelectricity inside this material system via a theoretical model. With these understandings, BOS is expected to have potential for further exploration.

The pursuit of high quality and uniformity for materials is required for practical usage. However, some forms of materials can impede performance, such as polycrystalline and random nanostructures, due to inevitable grain boundaries and defects. Thus, epitaxy is adopted to obtain a high-quality film and improve its performance. In this work, an epitaxial BOS film is fabricated via pulsed laser deposition (PLD). (LaAlO_3_)_0.3_(Sr_2_TaAlO_6_)_0.7_ (LSAT) (*a* = *b* = *c* = 0.384 nm, cubic) substrate is selected due to its compatible lattice with BOS (*a* = *b* = 0.387 nm, *c* = 1.192 nm, tetragonal). With this advantage, the mysteries of this material system can be revealed deeply. The antiferroelectricity of BOS is observed through atomic-scale images and simulation. This offers an opportunity to develop several related applications, including AFeFET,^19,20^ energy storage devices,^21,22^ and microelectromechanical systems.^23,24^ Meanwhile, the interior physics of optoelectrical properties in BOS is also discussed in this work. The dynamics of the photogenerated carriers dominates the photoresponse performance. Thus, a series of optical measurements were conducted to reveal the characteristics. Finally, the optoelectronics based on BOS have been fabricated, showing an excellent photoresponse compared to other material systems, credited to their high crystalline quality. This work sheds light on a deeper understanding of the physical properties of BOS and develops a new fabrication method, broadening the potential for more practical applications.

## Results and Discussion

### Structural Characteristics of Epitaxial Bi_2_O_2_S Thin Film

**Figure 1:**
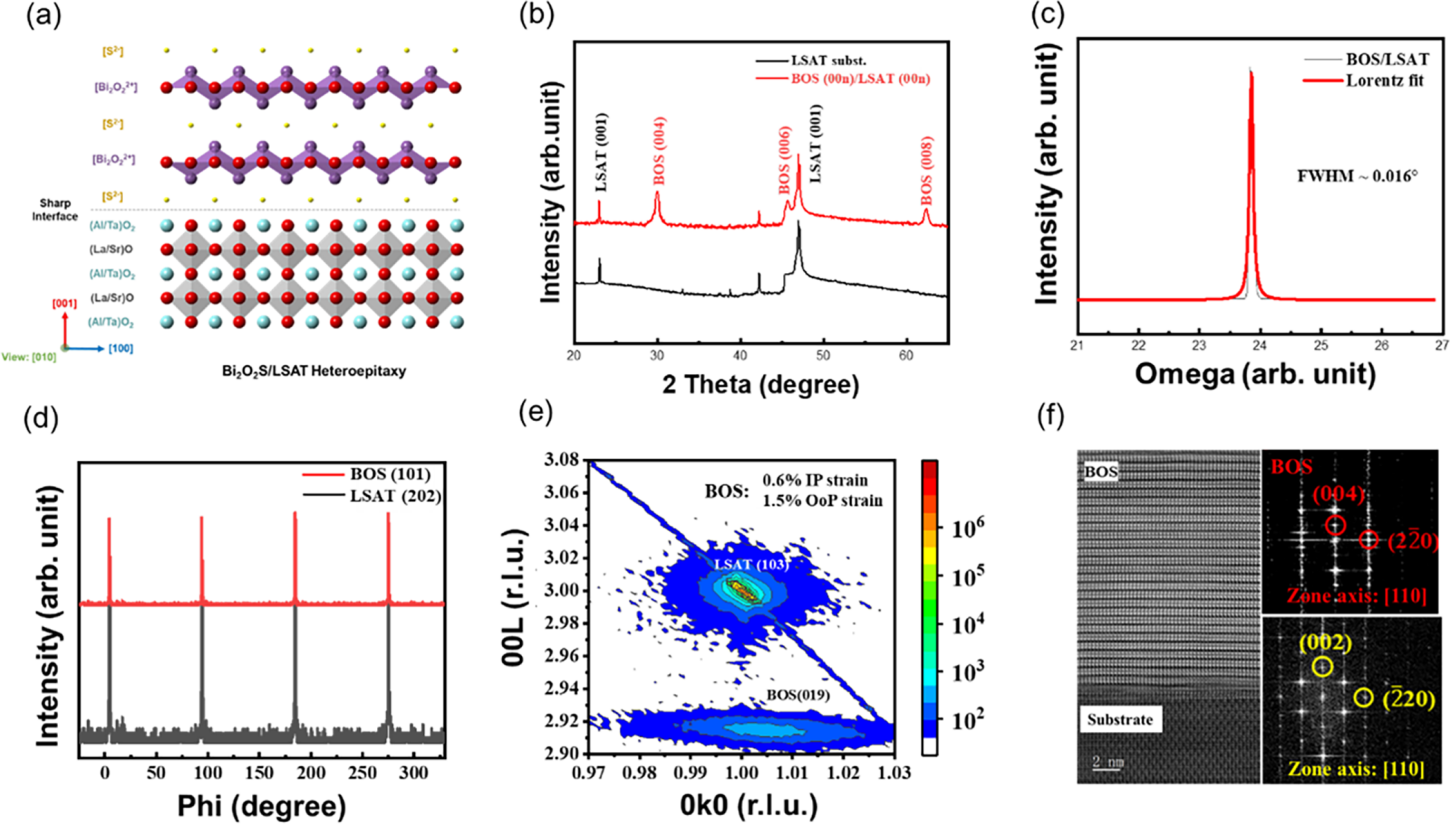
Structural information on the BOS/LSAT heterostructure. (a) Schematic diagram of the synthesized heterostructure. (b) Theta–2 theta scan of the BOS/LSAT heterostructure. (c) Rocking curve of BOS (060). (d) Phi-scan and (e) RSM mapping of the heterostructure. (f) Cross-sectional STEM images along with the FFT patterns.

First, the epitaxial characteristics of the BOS/LSAT heterostructure were studied by X-ray diffraction (XRD) (the existence of each element has been identified through the XPS measurements, Figure S1). Figure 1a shows the schematic diagram of the BOS/LSAT heterostructure. The compatible lattice between BOS and LSAT promotes the heterointerface quality during film stacking for subsequent measurements. To realize the crystalline orientation of the heterostructure, the θ–2θ scan shown in Figure 1b presents the pristine phase of the BOS and LSAT (001) substrate. Besides the LSAT (00L) signals, only the BOS (00L) series of signals appear without other secondary phases. Furthermore, the heterostructure performance strongly depends on the crystal quality. Thus, the rocking curve measurement of the BOS/LSAT heterostructure was conducted, and the results are shown in Figure 1c. The full width at half-maximum (fwhm) of BOS (006) peaks is ∼0.016°, indicating the high crystal quality of the heterostructure. Furthermore, the in-plane orientation is another crucial indication of the epitaxial nature. The phi-scan of the BOS/LSAT heterostructure is shown in Figure 1d. The 4-fold symmetry along (001) orientation is observed, and four sets of peaks at 90° intervals are displayed. It leads to the epitaxial relationship of the heterostructure as (002)BOS//(001)LSAT. After the realization of the fundamental understanding of the structure, the next objective is to acquire the lattice strain of BOS films on the LSAT substrate. The reciprocal space mappings (RSMs) were acquired at room temperature, as shown in Figure 1e. The result suggests 0.6% in-plane compressive and 1.5% out-of-plane tensile strains for the BOS layer. Such results indicate an influence of the substrate clamping effect. Furthermore, the microstructure of the whole heterostructure was investigated by scanning transmission electron microscopy (STEM). Figure 1f shows the cross-sectional high-angle annular dark field (HAADF) STEM image and the corresponding Fourier transform (FFT) diffraction patterns. The sharp interfaces between the BOS film and the LSAT substrate can be observed. Moreover, the reciprocal lattices in the FFT patterns of the BOS and LSAT layers are indexed in the insets, indicating the epitaxial relationship of BOS(002)//LSAT(001) and delivering consistent results with XRD. These efforts have established the correctness of phases and epitaxial relationships, providing the crystalline features.

### Local Centro-Asymmetry Structure

**Figure 2:**
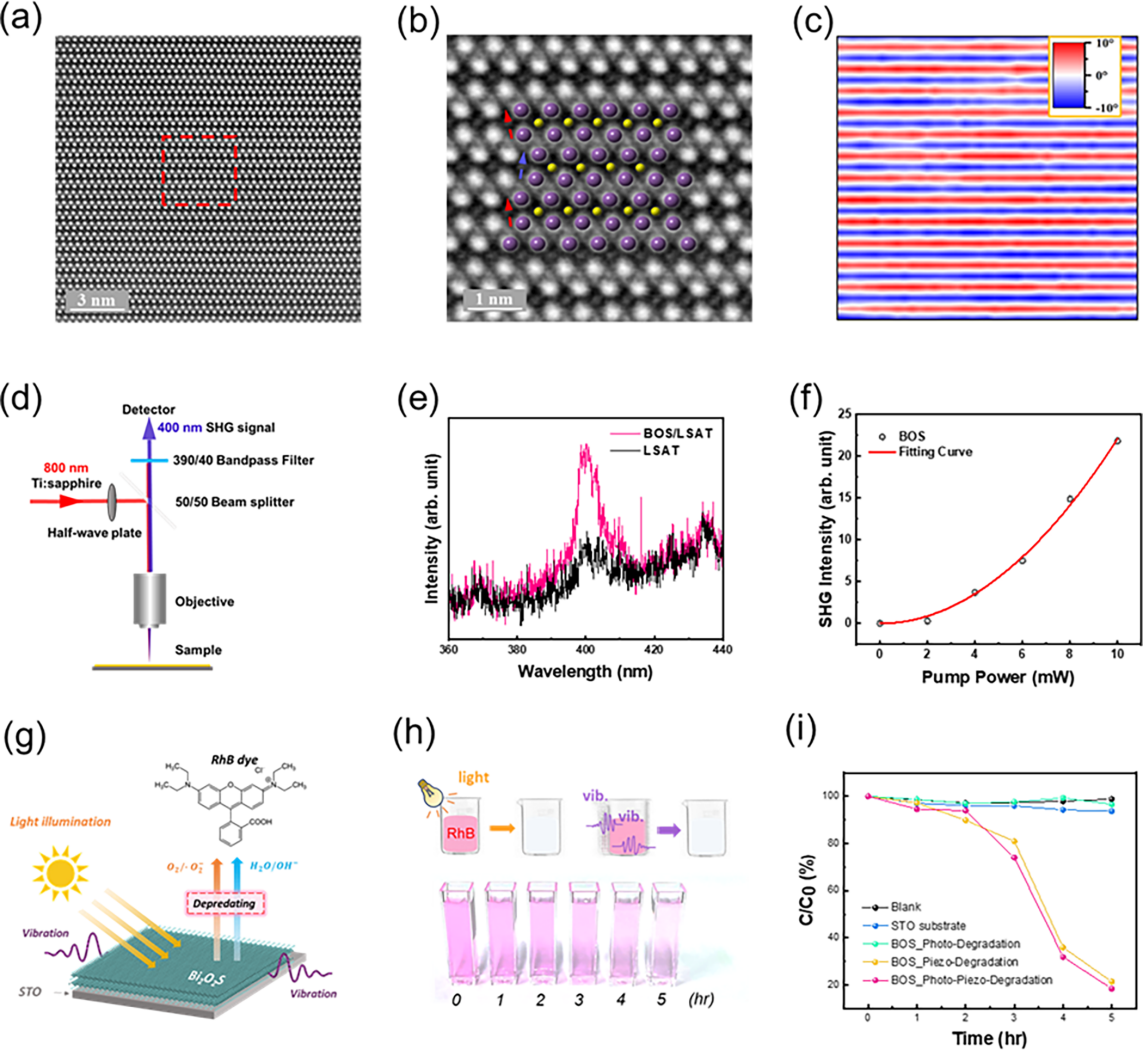
(a) Atomic-resolution HAADF-STEM images of the BOS layer. (b) High-magnification of the HAADF-STEM image corresponding to the area marked with a red dashed rectangle in (a). (c) Contour plot of the difference between the angles of the Bi parallelogram in the Bi–S–Bi layer and a right angle, calculated from (a) (red indicates that the calculated angle of the parallelogram is greater than 90°, while blue indicates that the calculated angle is less than 90°). (d) Schematic diagram of the SHG measurements. (e) SHG signals of the BOS film and LSAT substrate. (f) Pump power-dependent SHG intensity of the BOS film in (e) with a second-order fitting curve (solid red line). (g, h) Schematic diagram of the photodegradation, piezo-degradation, and photopiezo-degradation RhB dye degradation. (i) Results of RhB dye degradation, including the STO substrate for reference and the synthesized BOS film.

The previous section provides the results of structural characterizations of the BOS/LSAT heterostructure. Then, the detailed atomic arrangement of the heterostructure was characterized through HADDF-STEM. As shown in Figure 2a–c, we performed atomic-scale polarization characterization of the BOS film. Since the atomic intensity in the HAADF-STEM image is proportional to the atomic number *Z*^1.7^, the brighter spots in Figure 2a correspond to Bi atoms. In comparison, the darker spots correspond to the S atoms. Also, the EDS spectrum suggests the existence of each element in the synthesized sample (see Figure S2). A magnified view of the region outlined by the red dashed box in Figure 2a is shown in Figure 2b. It can be seen that the Bi and O atoms in each layer exhibit a certain amount of displacement along the in-plane direction, indicating that each [Bi_2_O_2_]^2+^ layer is ferroelectric, consistent with the literature reports.^18,25^ Furthermore, we found that the polarization directions of adjacent layers are opposite, indicating that multilayer BOS exhibits antiferroelectric ordering. Odd-numbered layers of BOS have a net polarization in the in-plane direction. To further verify the antiferroelectric characteristics of this material on a larger scale, CalAtom software [CalAtom: a software for quantitatively analyzing atomic columns in a transmission electron microscope image] was used to extract the positions of Bi atoms and calculate the angles of the parallelograms formed by Bi atoms in the Bi–S–Bi layers. The difference between the calculated angles and the right angle of 90° was plotted as a contour map, as shown in Figure 2c. Red indicates angles greater than 90°, and blue indicates angles less than 90°. The alternating layers with angles greater than and less than 90° demonstrate that the displacement directions of adjacent Bi_2_O_2_ layers are opposite, confirming the existence of BOS’s antiferroelectric ordering. Since the TEM results provide microscopic evidence for the local symmetry breaking, macroscopic characterization can be implemented to provide more insights. Symmetry-sensitive second-harmonic-generation (SHG) measurements were further carried out, and a schematic diagram of the SHG measurements is illustrated in Figure 2d. Figure 2e,f shows the spectrally resolved SHG intensity and power-dependent measurements. In Figure 2e, an obvious but weak SHG signal can be detected for the BOS film compared to the SHG signal from the substrate. Theoretically, the SHG signal can be expected from ferroelectric or uncompensated AFE ordering (odd layer). Thus, such a weak signal, together with the TEM image, verifies the existence of an odd-number AFE ordering.

According to this foundation, the local centro-asymmetry feature of BOS suggests the feasibility of piezophototronic and piezoelectric effects in the synthesized system. Thus, the RhB degradation experiment was further conducted since dye degradation could be driven by either solar energy or mechanical vibrational energy for photoactive semiconductors with a piezoresponse. The experiments were conducted under the three distinguished light illumination conditions (150 W Xe lamp), ultrasonic vibration (300 W, 40 kHz), and applied light with simultaneous ultrasonic vibration, respectively. Degradation activity (i.e., the concentration of RhB) was measured by the absorption spectrum using a UV–vis photospectrometer. The schematic diagrams of the photopiezo-degradation experiments are illustrated in Figure 2g,h. The ·O_2_^–^/·OH free radicals will be generated through light illumination or mechanical vibration, driving the reaction of RhB dye degradation. Consequently, the color of RhB dye will fade gradually with the reaction time. This color fading can be used to evaluate the concentration of RhB dye (Figure S3). Therefore, the photodegradation, piezo-degradation, and piezo-photodegradation of RhB by BOS film are presented in Figure 2i. The STO substrate is used for reference, ensuring that no other factors contribute to the experiments. Thus, the influence of the background is negligible. The results show that the reaction mainly originated from the piezoelectricity of the BOS film. There is nearly no response under the illumination. Such results further verify the piezoelectricity of the synthesized BOS film.

### Spectroscopic Analysis for the Band Structure and Carrier Lifetime

**Figure 3:**
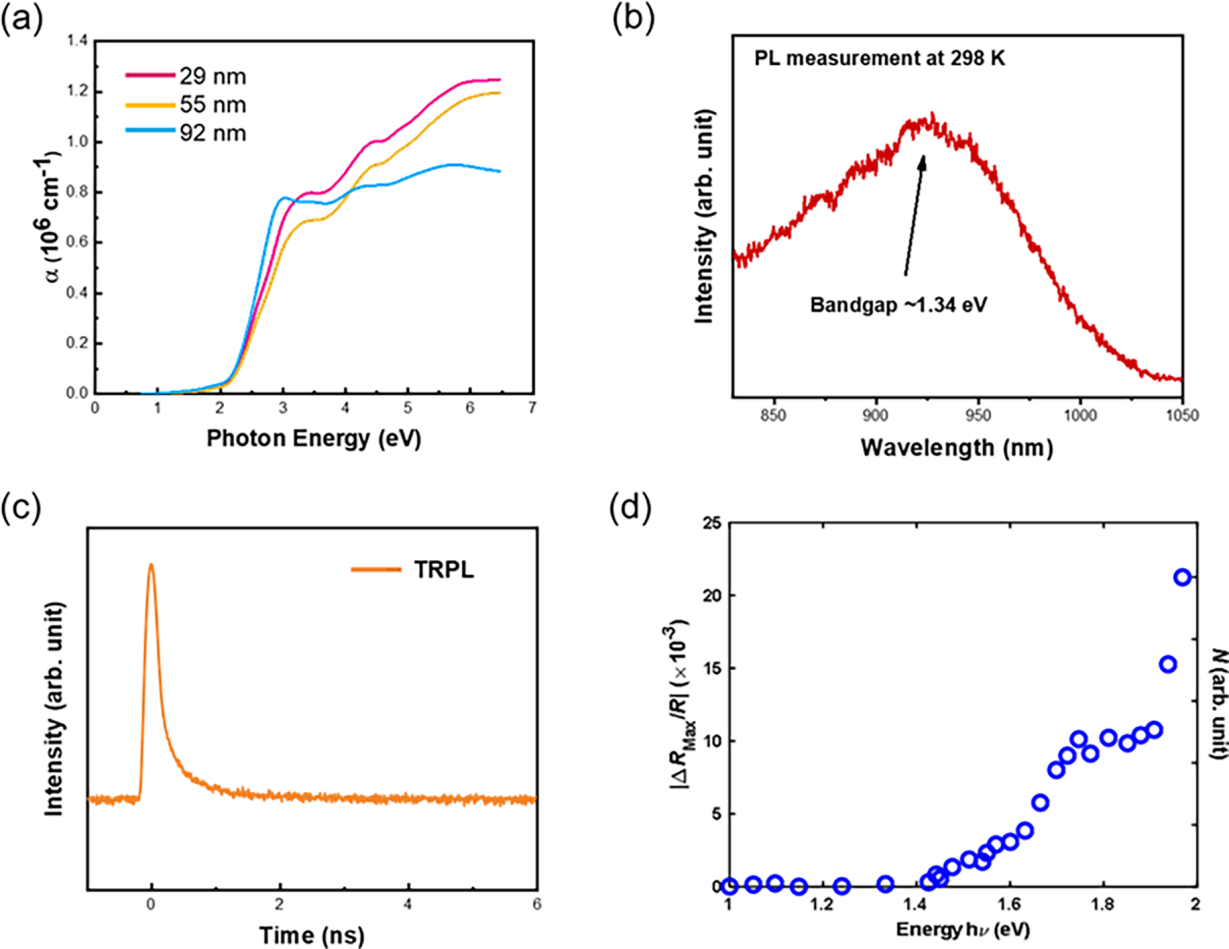
Spectrum analysis of the BOS/LSAT heterostructure. (a) In-plane optical absorption coefficient spectra of BOS with thicknesses of 29, 55, and 92 nm at room temperature. (b) PL measurement of the synthesized BOS film at room temperature. (c) The TRPL measurements are used to observe the carrier’s lifetime. (d) Result of the pump–probe measurement at room temperature.

After realizing the structural information on the synthesized epitaxial BOS/LSAT system, optical and electronic properties are further investigated. Figure 3a presents the room-temperature in-plane optical absorption coefficient (α) spectra of three BOS films through spectroscopic ellipsometric analysis. The obtained thicknesses of BOS are 29.0 ± 0.1, 55.0 ± 0.1, and 92.0 ± 0.1 nm, respectively. By plotting (α*E*)^0.5^ and (α*E*)^2^ against the photon energy, the indirect and direct band gap energies can be extracted using the Tauc relation.^26^ At room temperature, BOS films with thicknesses of 29, 55, and 92 nm exhibit indirect band gaps of 0.70 ± 0.02, 0.80 ± 0.01, and 1.18 ± 0.01 eV and direct band gaps of 0.96 ± 0.02, 1.12 ± 0.01, and 1.53 ± 0.01 eV, respectively. Furthermore, we conducted a photoluminescence (PL) measurement at room temperature to further determine the band gap of the epitaxial BOS film. Figure 3b shows the PL signal at 924 nm using a 532 nm laser. The extracted band gap is ∼1.34 eV, showing a consistent value with the results of the UPS/LEIPS measurements (Figure S4). With these efforts, the band structure of the epitaxial thin film has been identified through spectroscopic analysis.

On the other hand, the time-resolved PL (TRPL) spectrum was obtained to study the dynamics of the photogenerated charge carriers. Figure 3c shows the TRPL spectrum of BOS at room temperature. By extracting from the TRPL data, a double-exponential function is used for the fitting of the fluorescence attenuation curve:
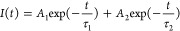
1where τ_1_ and τ_2_ are the emission lifetimes; *A*_1_ and *A*_2_ are the corresponding amplitudes. Meanwhile, the average decay lifetimes (τ_*A*_) are defined as
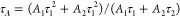
2

The calculation shows that the carrier lifetime is 0.1 ns at room temperature (Figure S5). However, compared to other material systems, the carrier’s lifetime at RT is relatively low, providing critical information for further photoresponse optimization. Finally, the pump–probe measurement was conducted to investigate the dynamics of the photoelectrons inside the BOS film, and Figure 3d displays a linear one-photon absorption spectrum. Based on the results, the maximum signal (Δ*R*_max_/*R*) occurs at near zero-time delay. This spectrum represents carrier densities *N* (*t* ≈ 0) (in an arbitrary unit) as a function of photon energies immediately following optical excitation.

### Photoresponse of the BOS/LSAT System

**Figure 4:**
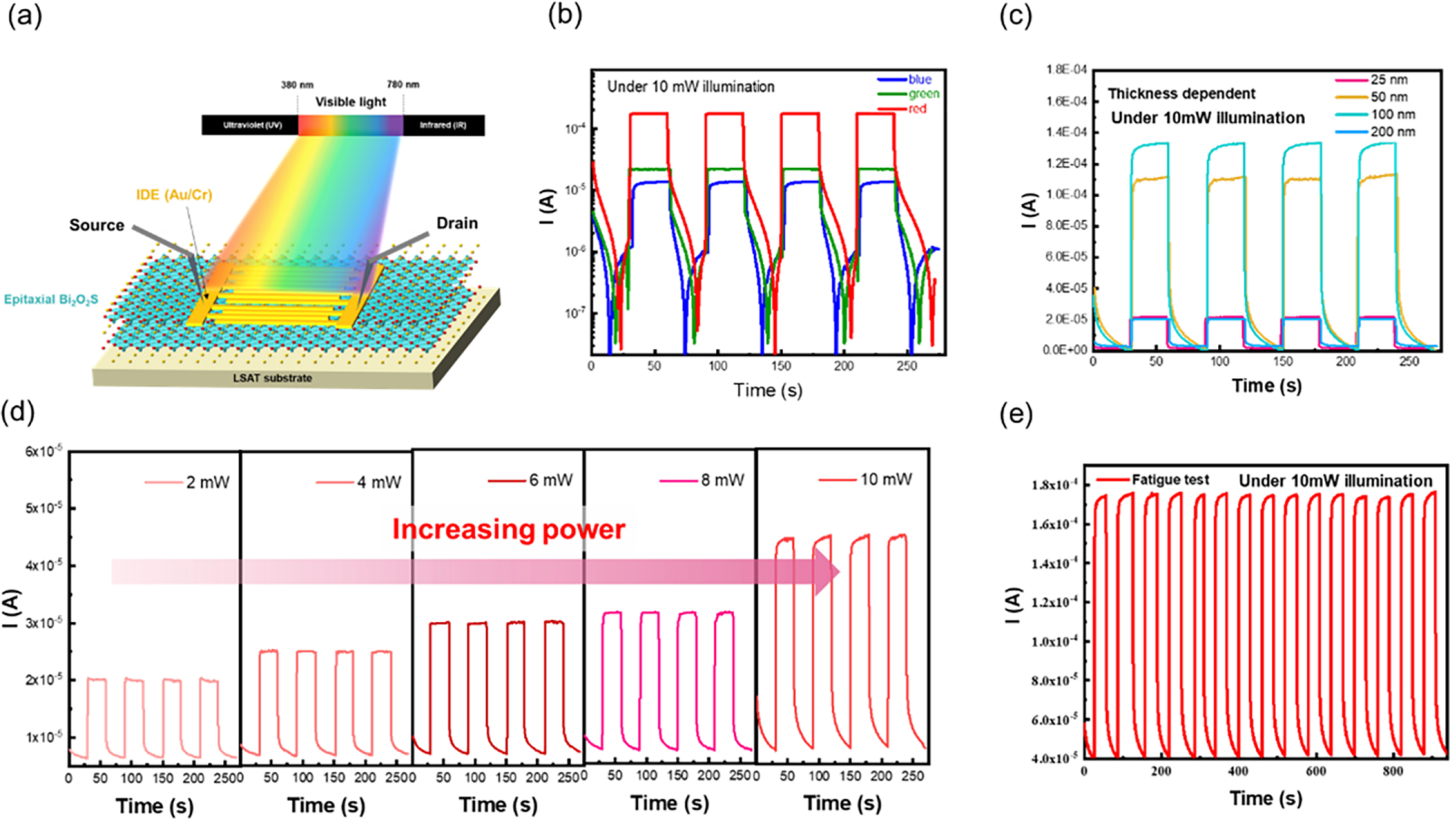
Photoresponsivity of the synthesized BOS/LSAT heteroepitaxy. (a) Schematic diagram of the *I*–*t* measurements. (b–d) Wavelength-, thickness-, and power-dependent *I*–*t* curves. (e) Result of the cycling test.

After a basic understanding of the band structure and optical properties of the BOS film, the photoresponsivity of the epitaxial BOS film is investigated. Figure 4a illustrates the layout of the photoresponse experiments. The wavelength in the visible light range (380–780 nm) is adopted for the measurement. Specifically, red, green, and blue lights are chosen to illuminate the sample. Figure 4b shows the photoresponse of BOS illuminated by different light sources. From the current–time (*I*–*t*) curves, the photoresponse triggered by the red light shows the highest on/off ratio (10^4^) compared to the others.^9−11,30,31^ Such a fact suggests better responsivity in the range of longer wavelengths. Consequently, the influence of the thickness parameter is then studied. The thickness-dependent (25, 50, and 100 nm) *I*–*t* curves have been conducted to optimize the value, as shown in Figure 4c. Here, the 100 nm-thick BOS shows the best responsivity under red-light illumination for about 61.14 mA/W, and the corresponding photodetectivity is 3.45E11, revealing a positive correlation with thickness. When the thickness of the BOS film is below 100 nm, the number of absorbed photons is limited, leading to a relatively low photocurrent. Conversely, the penetration depth of the light source should not exceed 100 nm. As a result, a decrease in the photoresponse is observed in the 200 nm thick BOS film. Moreover, the power-dependent *I*–*t* curves are also investigated, as presented in Figure 4d. The results show a higher responsibility for the illuminating power. Under higher power illumination, more carriers can be generated, leading to a higher photocurrent (the state of the art can be seen in Table 1). Based on the comparison, the photoresponse of BOS in the range of visible light reported in this work is better compared to others. Such a higher response is attributed to the excellent crystallinity of the synthesized film. In most instances, the photoresponse decreases significantly as the temperature rises due to improved carrier recombination and phonon scattering. However, the results indicate an increasing photocurrent with rising power. These findings suggest that the photoresponse primarily depends on the light intensity rather than being affected mainly by temperature. Finally, the stability of the photoresponse is crucial to be investigated. Figure 4e shows the result of the cycling test for the 100 nm-thick BOS’s photoresponse. According to the result, the on/off ratio is nearly unchanged (<1%) in 1200 s. This demonstration shows the high stability of the BOS’s photoresponsivity, invoking massive potential for further applications related to photoelectronic devices.

**Table 1:** Comparison of the Photoconduction in the Various BOS-Related Systems

material	form	wavelength (nm)	responsivity (mA/W)	response time (s)	reference
Bi_2_O_2_S	epitaxial film	620	61.4	2.00 × 10^–01^	this work
Bi_2_O_2_S	nanosheet	532	52	5.00 × 10^–01^	(11)
Bi_2_O_2_S	nanosheet	white	0.01	4.50 × 10^–02^	(10)
Bi_2_O_2_S		532	40		(31)
Bi_2_O_2_S	nanosheet	NIR (800–2500)	4000	1.00 × 10^–01^	(9)
Bi_2_O_2_S/GO	composite	532	0.00343	4.20 × 10^–01^	(30)

### Integration with Si Substrate

**Figure 5:**
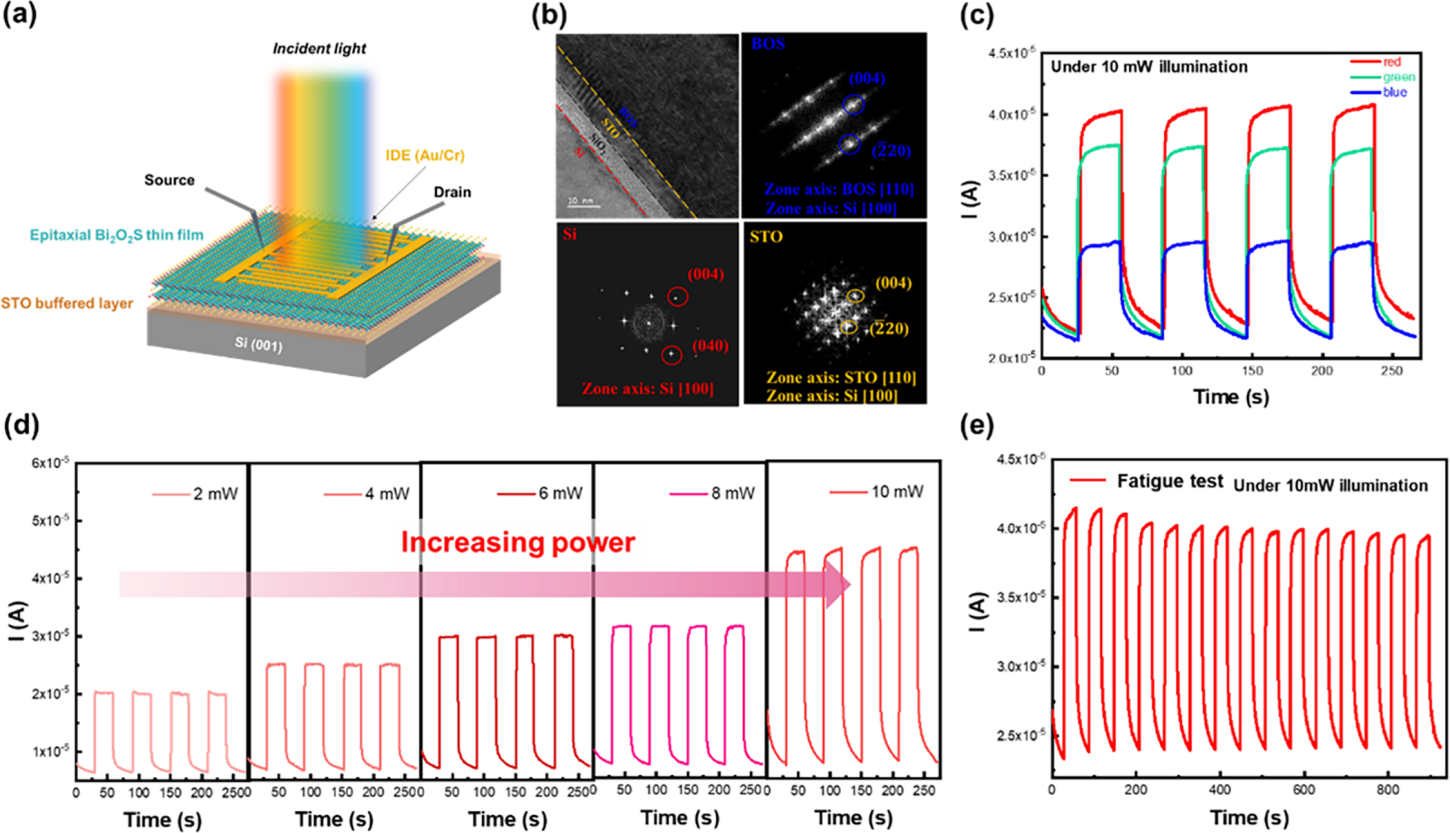
Structural characterization and photoresponsivity of the synthesized BOS/STO/Si heteroepitaxy. (a) Schematic diagram of the *I*–*t* measurements. (b) Cross-sectional TEM images. (c) *I*–*t* curves under illumination with different wavelengths. (d) Wavelength and power-dependent *I*–*t* curves. (e) Result of the cycling test.

The synthesized epitaxial BOS film presents an ultrahigh crystalline quality and an obvious photoresponse in the range of visible light. To extend the application of this material, silicon substrate integration is crucial. Figure 5a illustrates a schematic diagram of the BOS/STO/Si heterostructure. The buffered STO layer provides an opportunity for the BOS epitaxial growth. Consequently, the cross-sectional TEM images were captured along with the FFT patterns, as shown in Figure 5b. The results show the clear interfaces between the BOS film and STO/Si substrate (see Figure S6). Meanwhile, the reciprocal lattices in the FFT patterns of BOS, STO, and Si in the insets are indexed, showing single-crystalline-like patterns. The planes along the normal direction for each layer show the same orientation, while the zone axis presents a 45-degree deviation. XRD and phi-scan confirm this in-plane lattice compatibility between Si, STO, and BOS, where the epitaxial relationship can be identified as BOS(001)||STO(001)||Si(001) and BOS(100)||STO(100)||Si(110). Such a condition can be explained by the lattice compatibility for Si and (STO & BOS), consistent with the results from the phi scan (see Figure S7).

After the structural information is identified, attention is paid to studying the photoresponse of the BOS/STO/Si heteroepitaxy. Figure 5c shows the 100 nm-thick BOS photoresponse on the STO/Si substrate illuminated by different light sources. From the I-t curves, the photoresponse triggered by the red light still performs better than the others. The results indicate that the highest response wavelength will not be affected by the substrate effect. The values of the photoresponsivity of BOS on LAST and STO/Si are 61.14 and 14.22, respectively. This difference is attributed to the crystallinity of the STO/Si substrate being worse than pure LSAT substrate. Thus, the crystalline quality and photoresponse of BOS on LSAT are better. Moreover, the power-dependent *I*–*t* curves were also conducted, as shown in Figure 5d. A rising responsivity, along with increasing illuminating power, can be observed. Higher photocurrents attributed to more generated carriers can be measured. Finally, the fatigue test of the photoresponse is also studied. The result of the cycling test for the photoresponse is presented in Figure 5e. The on/off ratio shows excellent stability (<1%) in 1200 s. With these efforts, integrating BOS with Si substrate shows an epitaxial relationship and stable photoresponse. This invokes several potential applications for the 2D bismuth oxysulfide.

## Conclusions

This work presents the structural and optical properties of an epitaxial BOS film. The synthesized BOS/LSAT heterostructure shows a pure phase and excellent crystallinity (fwhm ≈ 0.016). Meanwhile, the epitaxial feature is confirmed through a series of structural characterizations. Moreover, the atomic-scale observation provides another insight into verifying the local centro-asymmetry of the BOS structure. The SHG and RhB dye degradation measurements can identify the piezoelectricity. After that, the optical spectra can reveal the BOS’s electronic structure. Similar band gap values can be extracted after these two measurements, leading to the construction of the band structure of the synthesized BOS film. Next, the BOS photoresponse is investigated from various aspects. First, the best photoresponse range at red light (∼700 nm) can be realized. Moreover, the optimized thickness (100 nm) and the stability of the photoresponse are also presented, suggesting robust performance for further applications. Finally, the demonstration of an integration with a Si substrate offers an opportunity for further practical applications. In conclusion, this study provides information on the epitaxial BOS thin film. Moreover, the intriguing physical mechanism of the interior and the device’s performance are also discussed. These efforts pave the way for studying this novel material system, invoking huge potential for next-generational electronic devices.

## Methods

### Sample Preparation

The BOS/LSAT heterostructure was fabricated via PLD with a commercial BOS target on commercial LSAT single-crystalline substrates. The vacuum chamber was evacuated to a pressure of 1 × 10^–6^ Torr before deposition. The BOS layer was grown on the LSAT substrate at 450 °C under 50 mTorr of O_2_ pressure. Lastly, the cooling process was conducted with a cooling rate of 0.3 °C/s. As for the BOS/STO/Si samples, the BOS film was deposited on the commercial STO/Si with the same oxygen pressure and substrate temperature. However, the difference in the growth parameter is at the cooling rate, which should be lowered to 0.1 °C/s.

### XRD Structural Characterization

The crystal information on BOS/LSAT and BOS/STO/Si heterostructures was identified by a high-resolution X-ray diffractometer (Bruker D8 Advance) with Cu K_α1_ radiation (λ = 1.54 Å). The θ–2θ scans of the heterostructures were recorded at a scan rate of 3°/min. The RSMs were recorded by a series of θ–2θ scans with different ω offsets and plotted in reciprocal lattice units normalized to the substrate lattice parameters.

### Photoresponse Measurements

Before the measurements, the Au/Cr top electrode (see Figure S8) was fabricated into interdigitated array electrodes using the E-gun process. The photoresponse of the BOS thin film was conducted in a B1500A semiconductor component parameter analyzer (*I*–*t* and *I*–*V* modes). The wavelengths of the incident light sources can be selected as blue (∼450 nm), green (∼550 nm), and red (∼600 nm). Meanwhile, the illumination power can be raised from 2 to 10 W.

### XPS/UPS/LEIPS Measurements

An ultraviolet photoelectron spectroscopy (UPS) and low-energy inversed photoelectron spectroscopy (LEIPS) are equipped on an X-ray photoelectron spectroscopy (XPS, PHI 5000 VersaProbe III, ULVAC-PHI) system, sharing the same ultrahigh vacuum chamber with a pressure below 10^–7^ Pa, to investigate the *in situ* band structure of the samples. For UPS measurements, helium-I UV-light (He–I, *hv* = 21.2 eV) is used as an incident source, and a −5 V bias was applied to the samples to enhance the photoelectron signal from the valence band. A pass energy of 2.6 eV, a scanning range of −7.0–16.6 eV, an energy step of 0.01 eV, and a time step of 20 ms were also set. Regarding LEIPS measurements, the low-energy electron gun (≤5 eV) was utilized, and the specimen was placed inside the vacuum chamber. In contrast, outside the optical lens, a bandpass filter (BPF= 4.88 eV), photomultiplier, and photodetector collecting near-ultraviolet (NUV) light were installed.^32^ The detection range, energy step, and time step are −9 to −2 eV, 0.04 eV, and 500 ms, respectively. The emitted photons from the sample were documented as a function of incident electron energy to create LEIPS spectra, and the current passed through the specimen and holder was recorded by a pico-ammeter to form LEET spectra. Detailed information about the conduction band can be further analyzed by combining these two spectra. Moreover, a conventional ultraviolet–visible spectroscopy (UV–vis, UV-2600i, SHIMADZU) was also conducted with a detecting wavelength of 200–900 nm to confirm the results from UPS/LEIPS measurements.

### Transmission Electron Microscopy Characterization and Analysis

TEM samples were prepared on a dual-beam FIB system (Helios G4 UX, FEI, USA). HAADF-STEM images and EDS mapping were acquired on a JEOL ARM300, capable of recording high-resolution STEM images with a spatial resolution of 63 pm. The microscope was equipped with a double spherical aberration (CS) corrector and an X-ray energy dispersive spectrometer (JED-2300 Series) with two 158 mm 2 mm silicon drift detectors (SDD).

### Spectroscopic Ellipsometer Measurements

Spectroscopic ellipsometer measurements are conducted at room temperature using an M-2000U ellipsometer from J. A. Woollam Co. The measurements cover a spectral range of 0.73 to 6.42 eV and are performed at incident angles of 60 and 70°. In spectroscopic ellipsometry, two parameters, Ψ and Δ, are measured.^26−29^ Ψ represents the amplitude ratio, while Δ indicates the phase difference of the reflected lights for incident p- and s-polarized lights on the material’s surface. For the analysis, the raw parameters of Ψ and Δ for the LSAT substrate are measured, and its complex optical constants are derived. These derived complex optical constants are then used in a stacked-layer model for spectroscopic ellipsometry analysis. This model, which includes a substrate, a thin film, a rough surface, and an air-ambient structure, is used to fit the raw Ψ and Δ parameters.

### Linear One-Photon Absorption Spectrum Measured by Pump–Probe Experiments

Nondegenerate pump–probe measurements were conducted to obtain a linear one-photon absorption spectrum at room temperature. The experiment utilized a laser amplifier system with a center wavelength of 1030 nm, a pulse duration of ∼200 fs, and a repetition rate of 5 kHz. The pump photon energies, ranging from 1.0 to 1.97 eV, were adjusted using an optical parametric amplifier. We measured the change in optical reflectivity, Δ*R*(*t*)/*R,* as a function of pump–probe time delay *t* at 0.86 eV where absorption is minimized, to isolate the contribution from refractive index changes. The probe beam has a spot size of ∼50 μm, with the pump beam size being at least two times larger for any pump photon energy. The pump power was controlled to maintain a consistent incident fluence of ∼1.21 mJ/cm^2^, which was confirmed to fall within a linear absorption range and not induce any measurable signal from the substrate.

## Data Availability

All the data needed to evaluate the conclusions in the paper are present in the paper and the Supporting Information.

## Supporting Information Available

Results of XPS characterization of BOS/LSAT; results of SEM-EDS of BOS/LSAT; absorbance of RhB dye varies along with time; results of the UPS/LEIPS measurements; fitting result of TRPL; EDS mapping of the cross-sectional TEM images; XRD of BOS/STO/Si heterostructure; and band diagram of Cr/BOS contact (PDF)
