# Supplementary material for: Epitaxial Antiferroelectric Bi2O2S Films with Superior Photoresponse
Source: ACS Appl Mater Interfaces. 2025 Mar 27;17(14):21392–400. doi: 10.1021/acsami.4c22419 (PMC11986903; doi:10.1021/acsami.4c22419)
Supplement: Supplementary file 1 — am4c22419_si_001.pdf [file am4c22419_si_001.pdf]

Supporting Information

Title: Epitaxial Antiferroelectric Bi_2O_2S Films with Superior Photoresponse

Yong-Jyun Wang[1], Chuan Chuang[1], Chia-Chen Chung[1], Po-Chih Chu[2], Wei-Chun Lin[2], Jian-Wei Zhang[3], Yu-Lun Chueh[1], Zhenzhong Yang[3], Rong Huang[3*], Keng-Hung Chang[4], Heng-Jui Liu[4], Hsiang-Lin Liu[5], Jia-Yuan Sun[6], Xin-Yun Chang[6], Hao-Che Chan[6], Chih-Wei Luo[6,7,8], Yu-Miin Sheu[6], Jyh-Ming Wu[1], Yi-Cheng Chen[1*], Ying-Hao Chu[1,6]*

Affiliation

[1]Department of Materials Science and Engineering, National Tsing Hua University, Hsinchu 300044, Taiwan

[2]Department of Photonics, National Sun Yat-sen University, Kaohsiung 804201, Taiwan

[3]Key Laboratory of Polar Materials and Devices (MOE) and Department of Electronics, East China Normal University, Shanghai 201203, China

[4]Department of Materials Science and Engineering, National Chung Hsing University, Taichung 402202, Taiwan

[5]Department of Physics, National Taiwan Normal University, Taipei 111396, Taiwan

[6]Department of Electrophysics, National Yang Ming Chiao Tung University, Hsinchu 300093, Taiwan

[7]Institute of Physics, National Yang Ming Chiao Tung University, Hsinchu 300093, Taiwan

[8]National Synchrotron Radiation Research Center, Hsinchu 300092, Taiwan

*Correspondence to: yhchu@mx.nthu.edu.tw, yicheng.chen@mx.nthu.edu.tw, and rhuang@ee.ecnu.edu.cn

The results of XPS characterization of BOS/LSAT

The XPS measurements of Bi, S, and O suggest the existence of these elements and the correct valance states. The O1s spectrum indicates the co-existence of two chemical states, Bi-O and S-O, in the BOS film. The valence states of Bi, Se, and O are +3, -2, and -2, respectively, consistent with the previous report. (33)

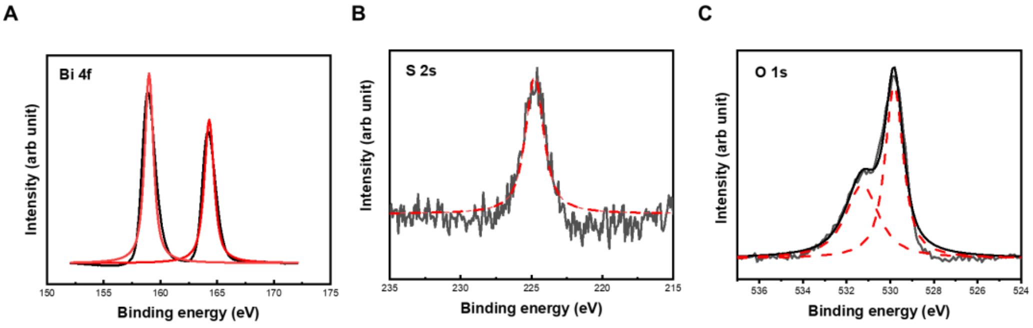

Figure S1 The XPS measurements of **A.** Bi, **B.** S, and **C.** O.

The results of SEM-EDS of BOS/LSAT

From Figure S2, it can be seen that the epitaxial growth of BOS films on the LSAT substrate results in a smooth surface. Furthermore, the EDS results confirm that Bi, S, and O elements are uniformly distributed, with no significant segregation observed.

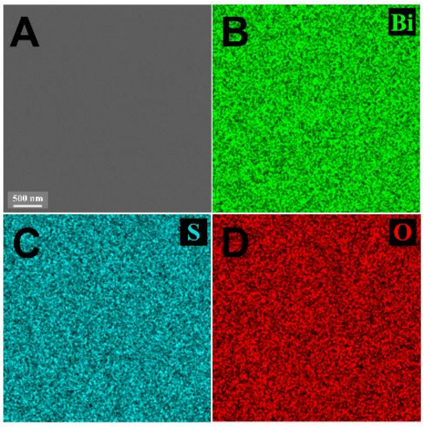

Figure S2. A. SEM image of the BOS. EDS elemental mapping of B**.** Bi, **C.** S, and **D.** O.

The absorbance of RhB dye varies along with time

From the results, we can observe barely any reaction for the photo-illumination due to the concentration of RhB dye showing no decline. However, a significant change can be observed when applying a mechanical vibration. These results imply the strong piezoelectricity of the synthesized BOS film.

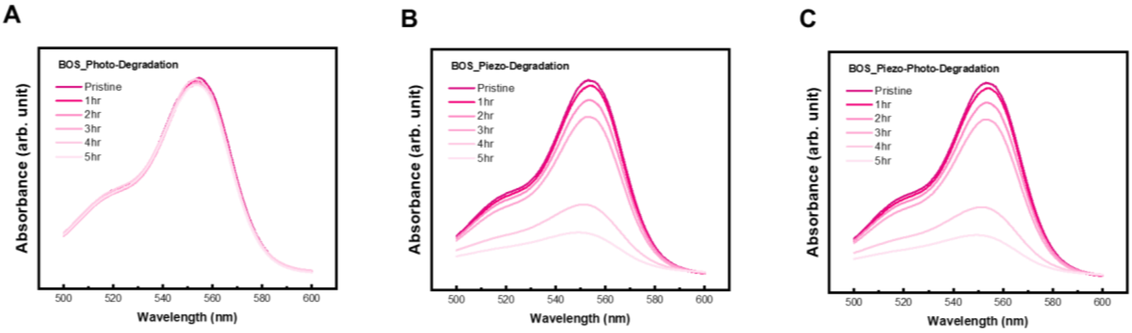

Figure S3 The absorbance of RhB dye for **A.** photo-illumination, **B.** mechanical vibration, and **C.** Photo-illumination + mechanical vibration along with time.

The results of the UPS/LEIPS measurements

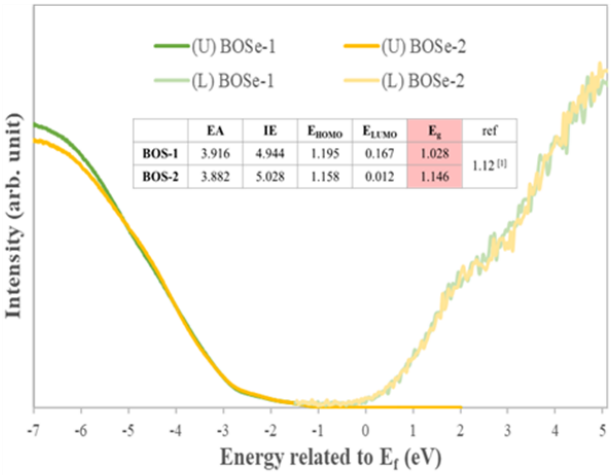

Figure S4. The UPS/LEIPS spectrum of the BOS/LSAT.

The fitting result of TRPL

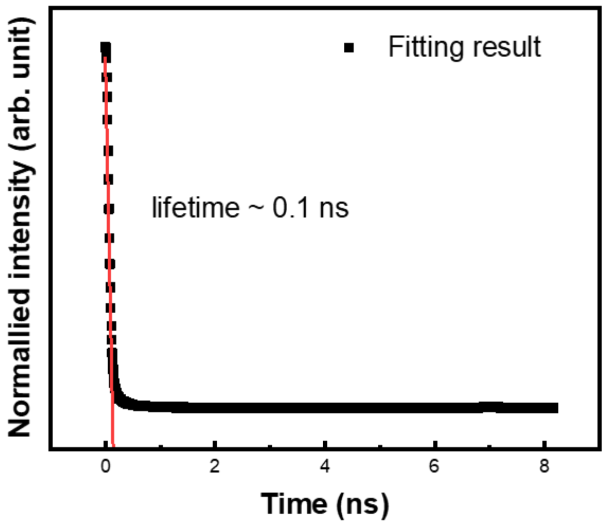

Figure S5. The fitting result of TRPL at room temperature.

Energy Dispersive Spectrometer (EDS) mapping of the cross-sectional TEM images

To distinguish each layer of the BOS/STO/Si heterostructure, EDS mapping of the cross-sectional TEM images is captured for identification. According to the results, the distribution of each element can be seen.

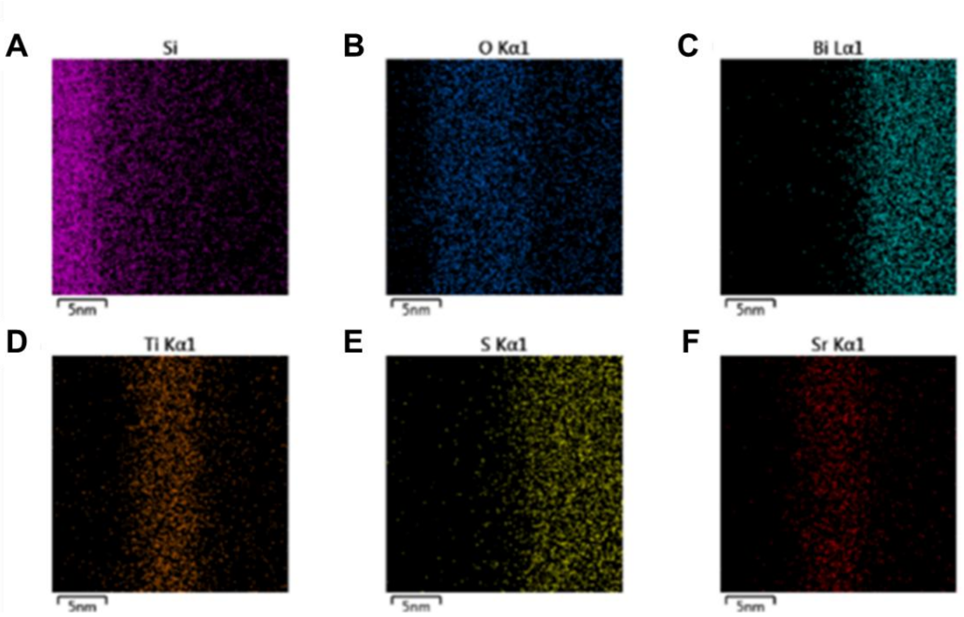

Figure S6 The EDS mapping of the cross-sectional TEM images

XRD of BOS/STO/Si heterostructure

XRD analysis is used here to identify the crystalline orientation of the BOS/STO/Si heterostructure. The theta-2 theta scan in S4A presents the pristine phase of BOS and STO/Si (001) substrate. Besides the substrate's signals, only (0K0) series signals of BOS appear without other non-pure phases (The peaks of STO cannot be observed due to the ultralow thickness ~1-2 nm). Meanwhile, the phi-scan of the BOS/STO/Si heterostructure is shown in Figure S6 B. The four-fold symmetry along (001) orientation is observed, and four sets of peaks at 90° intervals are displayed. The feature suggests that the BOS thin film is orderly arranged on the Si substrate, showing a successful integration.

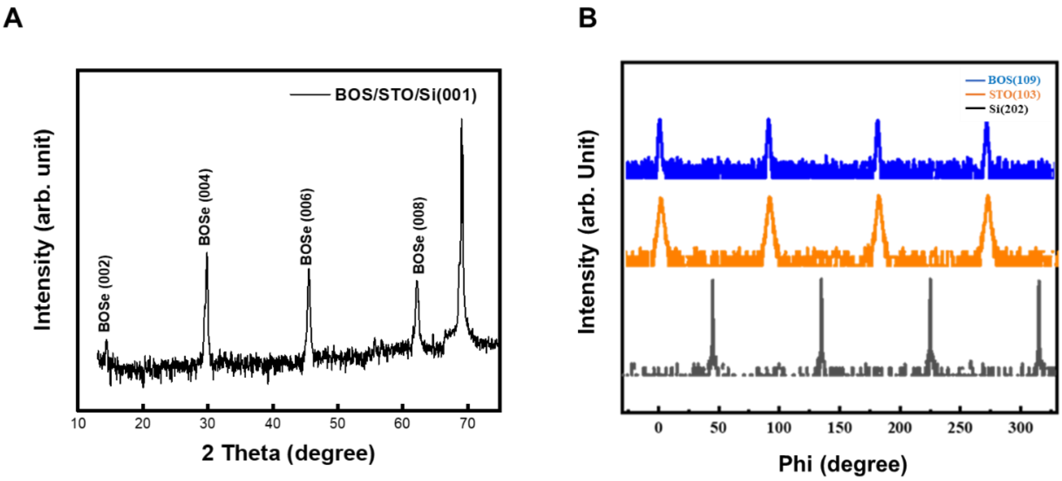

Figure S7 The XRD characterization of the BOS/STO/Si heterostructure. A. the theta-two theta scan and B. the phi scan of the synthesized heterostructure.

Band diagram of Cr/BOS contact

Cr electrode with work function for ~4.4 eV is adopted to be contacted with the BOS film (4.3 eV). With the compatibility of work function, some interface effects or Schottky contact can be prevented in this system.

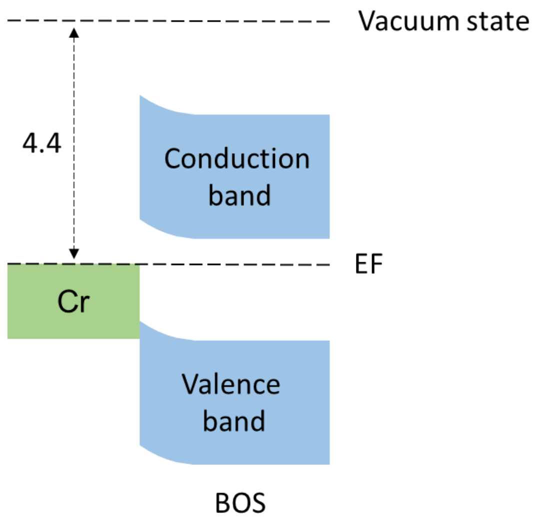

Figure S8. The band diagram of Cr/BOS film.
